# microRNA-342-5p and miR-608 inhibit colon cancer tumorigenesis by targeting NAA10

**DOI:** 10.18632/oncotarget.6458

**Published:** 2015-12-04

**Authors:** Hongju Yang, Qian Li, Jie Niu, Bai Li, Dejun Jiang, Zhihua Wan, Qingmei Yang, Fei Jiang, Ping Wei, Song Bai

**Affiliations:** ^1^ The First Affiliated Hospital of Kunming Medical University, Kunming, Yunnan, China; ^2^ Human Genetics Center of Yunnan University, Kunming, Yunnan, China; ^3^ Institute of Medicinal Biology Chinese Academy of Medical Science, Kunming, Yunnan, China

**Keywords:** NAA10, miR-342-5p, miR-608, colon cancer

## Abstract

miRNAs have been shown to play pivotal roles in the establishment and progression of colon cancer, but their underlying mechanisms are not fully understood. N-acetyltransferase NAA10 participates in many cellular processes, including tumorigenesis. Here we showed that miR-342-5p and miR-608 suppressed the tumorigenesis of colon cancer cells in vitro and in vivo by targeting NAA10 mRNA for degradation. Overexpression of miR-342-5p or miR-608 decreased NAA10 mRNA and protein levels and thereby suppressed cell proliferation, migration, and cell-cycle progression, as well as promoted apoptosis in SW480 and SW620 cells. More importantly, miR-342-5p and miR-608 significantly decreased the tumorigenic capacity of SW480 and SW620 cells in a mouse xenograft model. We also observed an inverse correlation between the expression of NAA10 and that of both miRNAs. Our results implicate miR-342-5p and miR-608 in colon cancer development and unveil the underlying mechanism of this phenomenon, which involves NAA10.

## INTRODUCTION

Colorectal cancer (CRC, including colon and rectal cancers) has a 5% overall incidence and is the third most common malignancy worldwide [1]. Although some progress has been achieved in the diagnosis and treatment of such diseases in the past decades, the 5-year survival rate remains at only 40-60% [2, 3]. The high incidence and poor prognosis of CRC imply that more efforts are needed to understand the underlying mechanisms of its formation and progression.

microRNAs (miRNAs) are endogenous 19-23 nt non-coding RNAs that modulate gene expression post-transcriptionally by either inducing mRNA degradation by targeting the 3′-untranslated regions (UTRs) of mRNA or inhibiting mRNA translation [4]. miRNAs have been shown to participate in a variety of physiological and pathological processes, including tumor formation and progression [5, 6]. In addition to activating oncogenic intracellular signaling pathways, such as phosphoinositide 3-kinase, epidermal growth factor receptor, transforming growth factor-b and Wnt pathways, miRNAs have been shown to play pivotal roles in the establishment and progression of CRC in past decades [7, 8]. miRNAs modulate CRC establishment, progression and prognosis most likely by influencing cancer stem-cell biology, angiogenesis and drug resistance, as well as epithelial-mesenchymal and mesenchymal-epithelial transitions [9]. There is a growing body of evidence showing that aberration in the expression of certain miRNAs leads to progression of CRC [10], however, the underlying mechanisms are not fully understood.

N-a-acetyltransferase 10 protein (NAA10), the paralog of the yeast gene arrest-defective-1 (ARD1), was first identified as the catalytic subunit of N-acetyltransferase A (NatA), a major N-terminal acetyltransferase complex in eukaryotes [11, 12]. Studies have shown that NAA10 is implicated in cell proliferation, metastasis, apoptosis, and autophagy [13-16]. Deficiency of NAA10 is embryonic lethal, and a single mutation in NAA10 results in a perinatal lethality (known as Ogden syndrome), due to reduced activity of the NatA complex [17]. In spite of lethality, other diseases related with NAA10 abnormality included cardiac arrhythmia, aged appearance, craniofacial abnormalities, hypotonia, and systemic lupus erythematosus [18, 19]. Recently, dysregulation of NAA10 has been reported to be associated with various human cancers [20], such as colorectal [21], breast [16, 22], lung [23], and prostate [24].

In contrast to the intensive studies on its functions, the regulation of NAA10 is largely unknown. In this study, we attempted to identify miRNAs regulating NAA10 expression and further demonstrated their roles in colon cancer tumorigenesis. Our results showed that two miRNAs, miRNA-342-5p and miRNA-608, targeted the 3′-UTR of NAA10 mRNA for degradation, suppressed cell proliferation and migration, and promoted apoptosis by downregulating NAA10 levels. In a mouse xenograft model, miR-342-5p and miR-608 efficiently repressed tumorigenesis of two colon cancer cell lines, SW480 and SW620. Accordingly, we observed reduced expression of miR-342-5p and miR-608 in patient-derived colon cancer samples, which was inversely correlated with the expression of NAA10. Our results suggest that NAA10 may serve as a potential target for colon cancer therapy, and miR-342-5p and miR-608 may have potential therapeutic applications in colon cancer patients.

## RESULTS

### Selection of miRNAs that target NAA10

**Figure 1:**
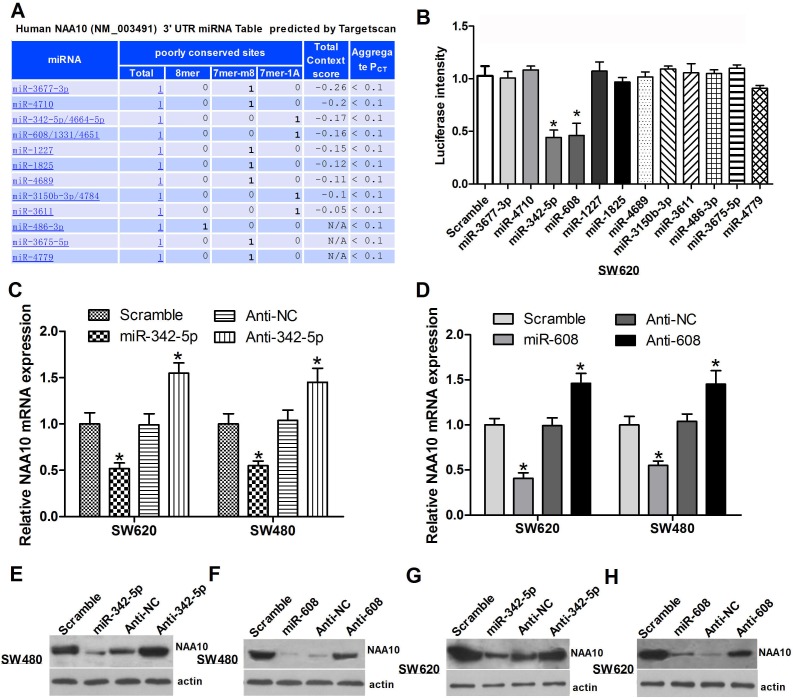
Selection of miRNAs targeting NAA10 **A.** Bioinformatics analysis predicted miRNAs regulating NAA10 are listed below. **B.** Luciferase activities in SW620 cells were measured 24 h after cotransfection of the candidate miRNAs and a NAA10-3′-UTR-containing luciferase vector. **C.**, **D.** SW480 or SW620 cells were transfected with the indicated miRNA mimics or anti-sense oligonucleotides (ASO), and 24 h later, NAA10 mRNA was extracted and analyzed by real-time PCR. **E.**, **F.**, **G.**, **H.** Cells were transfected as in **C.** and **D.** and lysed 36 h later to collect whole cell lysates (WCL) that were subjected to western-blot (WB) analysis. Experiments were repeated at least three independent times. Values represent the mean±s.d. *P < 0.05, calculated using Student's *t*-test.

A growing body of evidence shows that NAA10 plays pivotal roles in a variety of physiological and pathological processes; however, the miRNAs regulating NAA10 have not been reported. To identify miRNAs targeting NAA10, we first performed a bioinformatics analysis using TargetScan (Figure 1A). The candidates identified from this screen were then verified by luciferase assays. The candidate miRNAs and a luciferase vector containing the 3′-UTR of the NAA10 mRNA were cotransfected into SW620 cells. Out of 12 candidates, only miR-342-5p and miR-608 significantly suppressed the luciferase signal (Figure 1B), indicating that these two miRNAs specifically target the NAA10 3′-UTR. To further confirm these results, miR-342-5p and miR-608 antisense sequences were synthesized (referred to as anti-342-5p and anti-608). Overexpression of anti-342-5p significantly increased the mRNA level of NAA10 in both SW620 and SW480 cells (Figure 1C). Consistent with the NAA10 mRNA levels, NAA10 protein levels were reduced by miR-342-5p but increased by anti-342-5p in SW480 and SW620 cells (Figure 1E-1F). Similar results were observed with miR-608 and anti-608 (Figure 1D, 1G and 1H). These results implied that miR-342-5p and miR-608 may both target NAA10 for degradation and thereby repress the expression of NAA10.

### MiR-342-5p and miR-608 regulate NAA10 by directly binding to its 3′UTR

**Figure 2:**
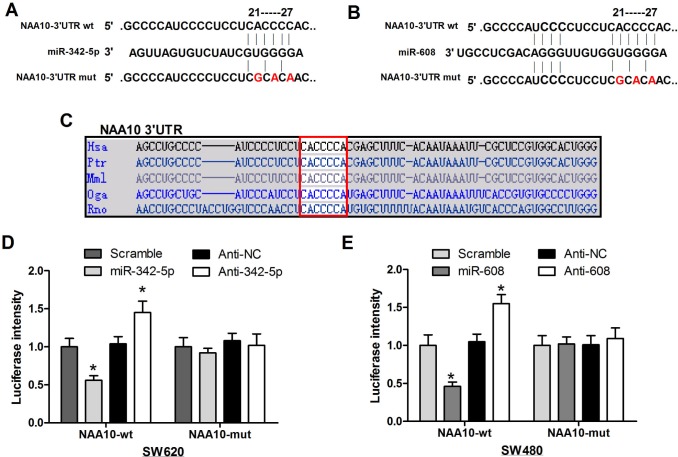
MiR-342-5p and miR-608 directly targeted NAA10 for degradation **A.**, **B.** The mutant NAA10-3′-UTR luciferase vectors were constructed by mutating the conserved sequences targeted by miR-342-5p and miR-608 in the NAA10-3′-UTR luciferase vector as indicated. **C.** Sequence aliments across species in the 3′-UTR of NAA10. **D.**, **E.** wild-type or mutant NAA10-3′-UTR luciferase vectors were co-transfected with the indicated miRNA mimics or ASOs into SW620 **D.** and SW480 **E.** cells, and the luciferase activities were measured 24 h later. Experiments were repeated at least three independent times. Values represent the mean±s.d. *P < 0.05, calculated using Student's *t*-test.

Sequence alignment revealed that the target sequence of miR-342-5p and miR-608 is conserved across species (Figure 2C). To test the specificity of such regulation, the conserved target sequence CACCCC was mutated to CGCACA in NAA10 luciferase vectors (Figure 2A and 2B). In line with the previous results, miR-342-5p decreased the activity of the wild-type (WT) NAA10 luciferase reporter, which was rescued by anti-342-5p (Figure 2D left). Meanwhile, neither miR-342-5p nor anti-342-5p had an effect on the mutant NAA10 luciferase activity (Figure 2D right). Similarly, miR-608 and anti-608 affected the WT but not the mutant NAA10 luciferase activity (Figure 2E). These results demonstrated that miR-342-5p and miR-608 repressed NAA10 by directly binding to its 3′UTR.

### MiR-342-5p and miR-608 repressed the tumorigenesis of colon cancer cells *in vitro*

**Figure 3:**
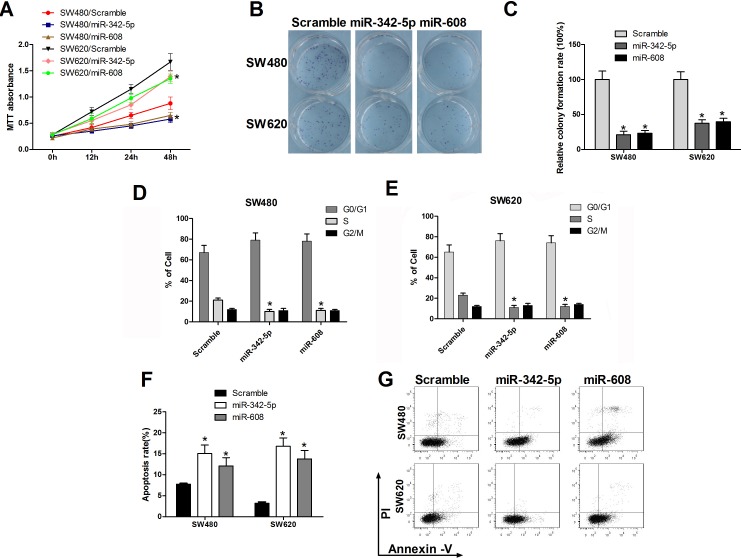
MiR-342-5p and miR-608 suppressed cell proliferation, migration, and colony formation and promoted apoptosis in colon cancer cells SW480 or SW620 cells were transfected with either the scramble or the indicated miRNA mimics. **A.** Cell viability was measured using MTT assays at the indicated time points. **B.**, **C.** Cells were seeded into a six-well plate after transfection, and the colonies were counted under a microscope 10 d later. **D.**, **E.** SW480 and SW620 cells were collected 24 h after transfection and then subjected to flow cytometry analysis. **F.** SW480 and SW620 cells were serum-starved overnight 24 h after transfection and then subjected to flow cytometry analysis **G.**. Statistical results are shown. Experiments were repeated at least three independent times. Values represent the mean±s.d. *P < 0.05, calculated using Student's *t*-test.

We have previously verified miR-342-5p and miR-608 target NAA10. Given that NAA10 is involved in multiple cellular processes, such as cell proliferation, migration, cell-cycle progression, we investigated whether miR-342-5p and miR-608 have roles in these cellular processes. Overexpression of miR-342-5p or miR-608 reduced the proliferation of SW480 and SW620 cells (Figure 3A). To further test the effects of miR-342-5p and miR-608 on tumorigenesis *in vitro*, we seeded a single cell in 6-well-plates and observed that cells transfected with miR-342-5p or miR-608 formed fewer colonies than cells transfected with a scramble miRNA control sequence (Figure 3B, 3C). Next, we examined the effects of the two miRNAs on cell-cycle progression. The percentage of cells in S-phase and G2/M-phase declined when cells were transfected with miR-342-5p or miR-608, respectively, in both SW480 (Figure 3D) and SW620 cells (Figure 3E). Lastly, we tested whether miR-342-5p or miR-608 overexpression affected cell survival. Cells transfected with either scramble miRNA, miR-342-5p or miR-608 were serum-starved overnight, and then subjected to flow cytometry analysis. Annexin V staining increased in cells transfected with miR-342-5p or miR-608 compared with those containing the miR scramble sequence in both SW480 and SW620 cells (Figure 3F and 3G). Collectively, these results indicated that miR-342-5p and miR-608 repressed tumorigenesis of colon cancer cells *in vitro*.

### Anti-342-5p and anti-608 enhanced tumorigenesis of colon cancer cells *in vitro*

**Figure 4:**
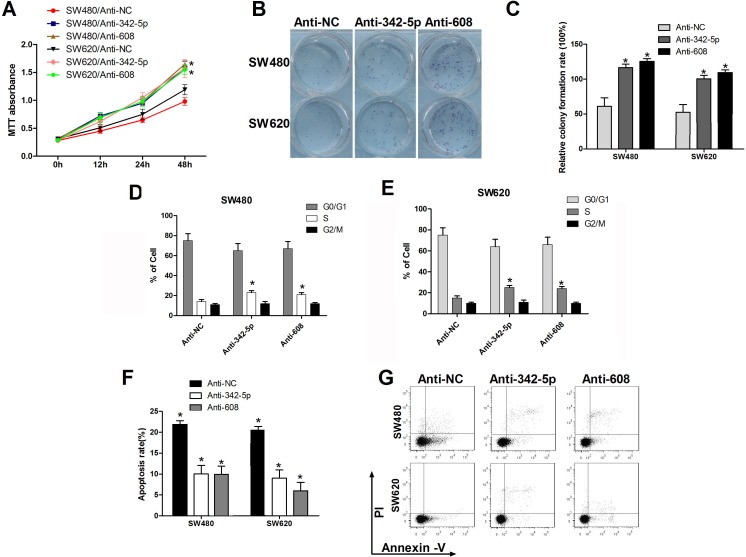
Anti-342-5p and anti-608 increased cell proliferation, migration, and colony formation and suppressed apoptosis in colon cancer cells SW480 or SW620 cells were transfected with either the negative control or the indicated ASO. **A.** Cell viability was measured using MTT assays at the indicated time points. **B.**, **C.** Cells were seeded into a six-well plate after transfection and the colonies were counted under a microscope 10 d later. **D.**, **E.** SW480 and SW620 cells were collected 24 h after transfection and then subjected to flow cytometry analysis. **F.** SW480 and SW620 cells were serum-starved overnight 24 h after transfection and then subjected to flow cytometry analysis **G.**. Statistical results are shown. Experiments were repeated at least three independent times. Values represent the mean±s.d. *P < 0.05, calculated using Student's *t*-test.

We have previously shown that miR-342-5p and miR-608 suppress cell proliferation, colony formation cell-cycle progression and promote apoptosis. Next, anti-342-5p and anti-608 were used to confirm these results. SW480 cells transfected with anti-342-5p or anti-608 exhibited enhanced proliferation compared with the negative control (Figure 4A). Similar results were observed in SW620 cells. Elevated colony formation abilities of these two cell lines were observed after transfected with anti-342-5p or anti-608 (Figure 4B, 4C). Overexpression of anti-342-5p or anti-608 resulted in increased percentage of SW480 and SW620 cells in S-phase and G2/M-phase (Figure 4D, 4E). Cells transfected with anti-342-5p or anti-608 were more sensitive to apoptosis induced by serum-starvation (Figure 4F and 4G). These results suggested that anti-342-5p and anti-608 promoted tumorigenesis of colon cancer cells *in vitro*, and therefore further confirmed our finding that miR-342-5p and miR-608 repressed tumorigenesis of colon cancer cells *in vitro*.

### MiR-342-5p and miR-608 repressed tumorigenesis of colon cancer cells *in vivo*

**Figure 5:**
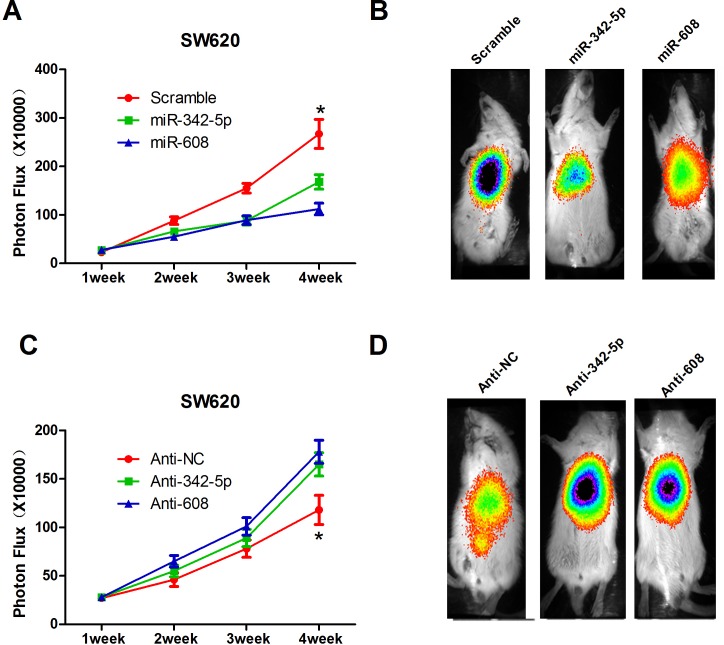
MiR-342-5p and miR-608 suppressed colon cancer cell tumorigenesis *in vivo* **A.** Cells expressing scramble, miR-342-5p or miR-608 were orthotopically injected into the colons of SCID mice (7 per group), and the tumor volumes were assessed by bioluminescence (BLI) measurements at the indicated time points. **B.** Representative BLI images of animals in **A.** 2 weeks after injection. **C.** Mice were orthotopically injected with cells expressing negative control, anti-342-5p or anti-608, and the tumor volume was assessed by bioluminescence (BLI) measurements at the indicated time points. **D.** Representative BLI images of animals in **C.** 2 weeks after injection. The color scale bar depicts the photon flux (photons per second) emitted from these mice. Values represent the mean±s.d. *P < 0.05, calculated using Student's *t*-test.

Previously, we showed that miR-342-5p and miR-608 repressed cell proliferation, migration, colony formation, and cell-cycle progression, and promoted apoptosis *in vitro*, suggesting that miR-342-5p and miR-608 may serve as tumor suppressors. To further validate this conclusion, stable cell lines expressing miR-342-5p, miR-608, anti-342-5p, and anti-608 were generated and tested in a mouse xenograft model. Data on tumorigenesis were collected at 1, 2, 3 and 4 weeks after implantation. Overexpression of miR-342-5p or miR-608 significantly restrained the tumor growth by 4 weeks (Figure 5A and 5B), while anti-342-5p and anti-608 promoted tumor growth (Figure 5C and 5D). In conclusion, our results suggested that miR-342-5p and miR-608 repressed tumor growth *in vivo*.

### MiR-342-5p and miR-608 suppress tumorigenesis by downregulating NAA10

**Figure 6:**
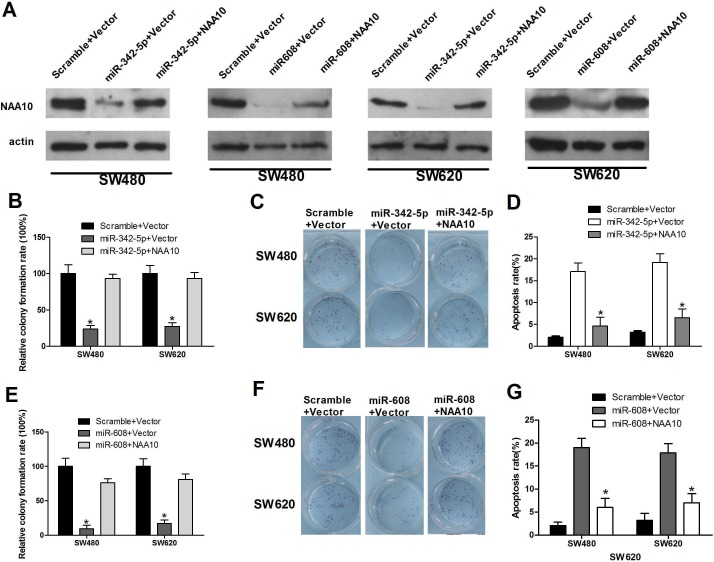
NAA10 restoration rescued miR-342-5p and miR-608 mediated cell proliferation, migration, and colony formation defects and promoted cell apoptosis suppressed by miR-342-5p or miR-608 SW480 or SW620 cells were co-transfected with the indicated miRNA mimics and vectors. **A.** 36 h after transfection, cells were lysed and WCL were subjected to WB analysis (β-actin as a loading control). **B.**, **E.** Single cells were seeded into 6-well plates 6 h after transfection, and the colonies were counted 24 h later. **C.**, **F.** SW480 (upper panel) and SW620 cells were seeded into the upper part of a transwell chamber before transfection, and 24 h later cells in the bottom were counted under a microscope after staining. **D.**, **G.** SW480 and SW620 cells were collected 24 h after transfection and then subjected to flow cytometry analysis. Experiments were repeated at least three independent times. Values represent the mean±s.d. *P < 0.05, calculated using Student's *t*-test.

NAA10 has been reported to regulate various cellular processes including cell proliferation [25], migration [26], apoptosis [14] and cell-cycle progression [27] in previous studies. We have demonstrated that miR-342-5p and miR-608, the regulators of NAA10, also play important roles in such cellular processes; however, the underlying mechanism is not clear. Thus, we investigated whether miR-342-5p and miR-608 regulate these cellular processes by modulating NAA10 levels. To verify this hypothesis, we co-transfected miR-342-5p and NAA10 into SW480 and SW620 cells. Consistent with our previous results, NAA10 protein levels declined when cells were transfected with miR-342-5p or miR-608 alone. Cotransfection of NAA10 with miR-342-5p or miR-608 restored NAA10 levels (Figure 6A). Overexpression of NAA10 rescued the inhibition of colony formation and migration induced by miR-342-5p transfection in SW480 and SW620 cells (Figure 6B and 6C). NAA10 restoration significantly reduced apoptosis induced by serum-starvation in cells transfected with miR-342-5p (Figure 6D). Additionally, NAA10 restored the reduction in colony formation and mobility (Figure 6E and 6F) as well as the elevated rate of apoptosis resulting from transfection of miR-608 (Figure 6G). Collectively, these results indicate that NAA10 can partially rescue colon cancer tumorigenesis that is repressed by miR-342-5p or miR-608 overexpression *in vitro*. Thus, miRNA-342-5p and miR-608 suppress colon cancer tumorigenesis most likely by downregulating NAA10.

### NAA10 is involved in colon cancer tumorigenesis *in vitro* and *in vivo*

**Figure 7:**
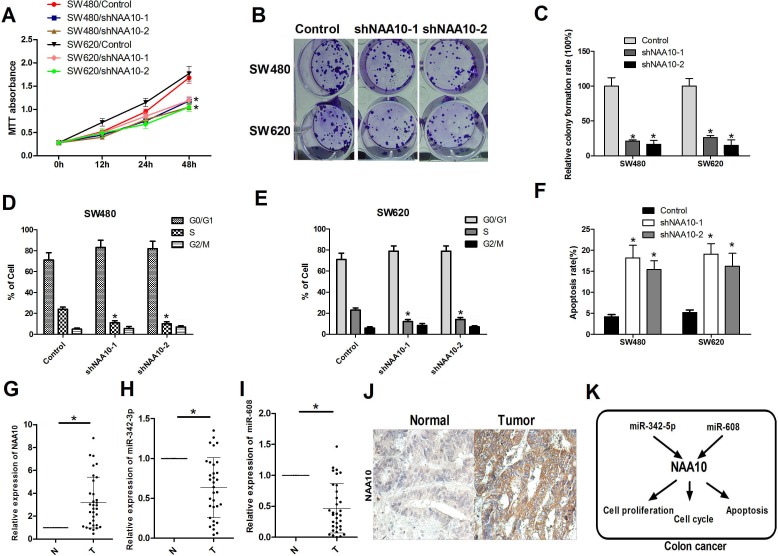
NAA10 participated in colon cancer tumorigenesis *in vitro* and *in vivo* **A.**, **B.**, **C.** SW480 and SW620 cells were transfected with control shRNA and NAA10 shRNA as indicated. Cell viability was measured by MTT assays and colony formation assays. **D.** and **E.** SW480 and SW620 cells were collected 24 h after transfection with control, shNAA10-1 and shNAA10-2 and then subjected to flow cytometry analysis. **F.** SW480 and SW620 cells were serum-starved overnight 24 h after transfection as in **A.** and then subjected to flow cytometry analysis. **G.**, **H.** and **I.** 33 paired human colon cancer samples and adjacent normal mucosa tissues were collected and total RNA was extracted. NAA10 mRNA, miR-342-5p and miR-608 levels were determined by real-time PCR. **J.** Sections from paired human colon cancer samples and adjacent normal tissues were subjected to IHC analysis. **K.** Model of the miR-342-3p and miR-608 regulate NAA10 in colon cancer cells. Experiments were repeated at least three independent times. Values represent the mean±s.d. *P < 0.05, calculated using Student's *t*-test.

Previous studies have shown that NAA10 is upregulated in colorectal cancer [21] and is associated with poor prognosis in colon cancer patients [28], however, the role of NAA10 in colon cancer development remains largely unknown. To investigate this question, two different shRNAs were designed to target NAA10, and both were found to efficiently decrease the expression of NAA10 in SW480 and SW620 cells. NAA10 knockdown significantly decreased the proliferation of SW480 and SW620 cells, similar to the effects of miR-342-5p and miR-608 (Figure 7A-7C, 7F). Knocking down NAA10 by shRNAs also inhibited cell-cycle progression as evidenced by a decrease in the percentage of cells in S and G2/M phases (Figure 7D, 7E). To further evaluate the possible role of NAA10 in colon cancer, we collected 33 pairs of human colon cancer samples and adjacent normal mucosa tissues. The mRNA levels of NAA10 were significantly higher in tumors compared with adjacent normal mucosa tissues (Figure 7G). These results were further confirmed by immunohistochemical analysis (Figure 7J). The inverse correlations between NAA10 and the two miRNAs miR-342-5p and miR-608 were observed in the paired colon cancer samples and adjacent normal mucosa tissues (Figure 7H, 7I). These results further confirmed that NAA10 is involved in colon cancer tumorigenesis, and miR-342-5p and miR-608 participate in colon cancer tumorigenesis by regulating NAA10 (Figure 7K).

## DISCUSSION

Previous studies revealed that NAA10 s associated with CRC and regulates various cellular processes including cell proliferation, cell-cycle progression, and apoptosis [14, 15, 21, 28]. NAA10 is increasingly recognized as an important regulator in multiple cellular processes and the development of a variety of cancers, but the regulatory mechanisms of such processes is largely unknown. In this work, we demonstrated that miR-342-5p and miR-608 directly targeted NAA10 mRNA and reduced the expression of NAA10, therefore inhibiting colon cancer tumorigenesis (Figure 7K).

Studies show that NAA10 is a stable protein that is expressed in a broad range of tissues and tumor cell lines [11, 29, 30]. However, besides its autoacetylation, the post-transcriptional regulation of NAA10 has not been reported [25]. Here, we identified two new miRNAs (miR-342-5p and miR-608) targeting NAA10 mRNA for degradation and suppressing its expression. MiR-342-5p and miR-608 can directly bind to the 3′-UTR of NAA10. Overexpression of miR-342-5p or miR-608 reduced NAA10 expression *in vitro* and *in vivo*. Conversely the ASOs of the two miRNAs increased NAA10 expression in two colon cell lines. To our knowledge, this is the first study showing that NAA10 is regulated post-transcriptionally by miRNAs.

MiR-608 was reported to suppress chordoma malignancy by directly targeting EGFR and Bcl-xL [31], but its role in other cancers is unknown. Our results have extended the tumor suppressive function of miR-608 to include colon cancer and have validated NAA10 as a new target of miR-608. Additionally, this is the first report that investigates the function of miR-342-5p. Although we have demonstrated that miR-342-5p and miR-608 suppresses tumorigenesis most likely by targeting NAA10, there may be other targets responsible for the effects of these two miRNAs, considering the low specificities of miRNAs. This possibility should be clarified in future works.

Though there is a growing body of evidence showing that NAA10 plays a pivotal role in cancer development [16, 23, 24], the function of NAA10 is controversial [32]. As an activator of β-catenin NAA10 promotes cell-cycle progression and therefore facilitates cell proliferation, suggesting that NAA10 may act as an oncogene [15]. However, there are reports showing NAA10 may repress tumorigenesis via two mechanisms: by decreasing cell proliferation and promoting autophagy by acetylating and stabilizing TSC2, a repressor of mTOR signaling, or by preventing tumor cell migration and invasion by acetylating and deactivating myosin light chain kinase (MLCK), a Ca^2+^/calmodulin-dependent protein kinase [16, 26]. Our results suggested that silencing NAA10 led to lower level of colon cancer cell tumorigenesis *in vitro*. Combined with the fact that NAA10 was upregulated in tumors, these data indicated NAA10 might act as an oncogene in colon cancer. Given the complexity of NAA10 regulation of cancer development, further studies are needed to uncover the molecular mechanisms behind how NAA10 controls colon cancer tumorigenesis.

## MATERIALS AND METHODS

### Human tissue samples

Thirty-three pairs of human colon cancer samples and adjacent normal mucosa tissues were obtained from The First Affiliated Hospital of Kunming Medical University. Detailed pathologic and clinical data were collected for all samples including Edmondson tumor grade, invasion and metastasis. The diagnoses of these samples were verified by pathologists. The collection of human tissue samples was approved and supervised by the Ethics Committee of The First Affiliated Hospital of Kunming Medical University.

### Mouse xenograft model

All animal work was conducted in accordance with a protocol approved by Ethics Committee of The First Affiliated Hospital of Kunming Medical University. For orthotopic implantation, 1×10^7^ viable scramble, miR-342-5p, or miR-608 expressing cells were injected into CB-17 SCID mice colons in a volume of 0.1 ml. Tumor growth were monitored by live animal BLI (Xenogen IVIS system) once per week.

### Cell culture, plasmids and transfection

The human SW480 and SW620 cells were cultivated in RPMI1640 Medium containing 10% fetal bovine serum plus 2 mM L-Glutamine. All cells were split prior to establishment of confluence and incubated at 37°C in a humidified incubator with 5% CO_2_. MiR-342-5p, miR-608, anti-342-5p and anti-608 were purchased from Shanghai GenePharma (Shanghai, China). NAA10 full length CDS was cloned into the pCMV2-myc vector. Lentiviral vectors for miR-342-5p, miR-608, anti-342-5p, anti-608 (40 ng, respectively) and their control vectors were purchased from Sigma. The shRNA targeting NAA10 (region 153-177, GGAGTTCCTGGTGTCGGCATTCTTA) was designed and synthesized by GenPharm (Shanghai, China) and cloned into the pGreenpuro vector (Invitrogen). An unrelated sequence was used as a negative control (provided by GenPharm). Transfections of miRNA mimics and ASOs were carried out using Lipofectamine-2000 (Invitrogen, Carlsbad, CA) according to the manufacturer's instructions.

### RNA preparation and quantitative PCR

RNA was extracted from cells or tissue samples using the mirVanamiRNA Isolation Kit (Ambion, USA) according to the manufacturer's instructions. Small RNA fractions (smaller than 200 nt) were separated and purified according to this procedure. cDNA was obtained using M-MLV (Promega, USA) and 1 μg of isolated RNA. The relative levels of miR-342-5p and miR-608 were detected by stem-loop RT-PCR with following conditions: denaturing the DNA at 94°C for 4 min followed by 40 cycles for amplification: 94°C for 60 s, 58°C for 60 s, 72°C for 60 s. U6 snRNA was used as an endogenous control. Quantitative PCR was performed on an ABI 7500 thermocycler (Applied Biosystems) using SYBR^®^ Premix Ex Taq^™^ Kits (Perfect Real Time, TaKaRa, Japan) according to the manufacturer's instructions.

### Detection of cell proliferation capacity

To determine the proliferative capacity of cells, growth curve and colony formation assays were employed. Cells were seeded in triplicate in 24-well plates at a density of 1×10^4^ SW480 or SW620 cells per well and were counted over a 6-day period beginning on the second day. Cells were harvested by trypsinization and counted four times per well. The growth curves over 6 days were drawn according to the mean values of the three wells. For colony formation assays, the number of viable cell colonies was determined 14 days after seeding SW480 or SW620 cells into 12-well plates. The colony formation ratio was calculated with the following equation: colony formation ratio (%) = (number of colonies/number of seeded cells) × 100.

### Fluorescent reporter assays

The human NAA10 3′UTR harboring three putative miR-342-5p and miR-608 target binding sequences was synthesized by GenPharm (Shanghai, China). Luciferase constructs were created by ligating the WT and mutant 3′UTRs downstream of the lucORF in the pMIR-REPORT luciferase vector (Ambion). For the fluorescent reporter assay, cells were seeded in a 48-well plate the day before transfection. The cells were co-transfected with miRNA mimics, ASOs, or their respective controls with WT or mutant NAA10-3′UTR. The cells were lysed 48 h later and the intensity of luciferase was detected.

### Western blotting

Western blotting was performed to determine protein expression of PREX2a and Akt. Cells were lysed in RIPA buffer to isolate total protein. β-actin was used as a loading control. The polyclonal rabbit anti-human NAA10 (ab155687, Abcam Inc., Cambridge, MA, USA), and anti-actin (ab151526, Abcam Inc., Cambridge, MA, USA) were used.

### Cell-cycle assay

Transfected SW480 and SW620 cells were seeded into 6-well plates for 24 h in complete medium before cells were deprived of serum for 48 h and then returned to complete medium for an additional 24 h. All cells were collected by centrifugation, fixed in 95% ethanol, incubated at −20°C overnight and washed with phosphate buffered saline (PBS). Then, cells were resuspended in 1 ml FACS solution (PBS, 0.1% TritonX-100, 60 ug/ml propidium iodide (PI), 0.1 mg/ml DNase free RNase, and 0.1% trisodium citrate). After a final incubation on ice for 30 min, cells were analyzed using a FACS Calibur flow cytometer (Beckman Coulter). A total of 10,000 events were counted for each sample.

### Apoptosis assay

Transfected SW480 and SW620 cells were seeded into 6-well plates for 24 h in complete medium and then serum-starved for 8 h. All cells were collected by centrifugation and then stained with 0.5 μg of annexin V for 20 min in the dark and with 10 μg of PI for 5 min. Cells were analyzed using a FACS Calibur flow cytometer (Beckman Coulter). A total of 10,000 events were counted for each sample.

### Statistical analysis

Student's tests were performed to analyze the significance of differences between sample means obtained from three independent experiments. Differences were considered statistically significant at p < 0.05.
